# Secondhand Smoke Exposure and Depressive Symptoms among Korean Adolescents: JS High School Study

**DOI:** 10.1371/journal.pone.0168754

**Published:** 2016-12-30

**Authors:** Na Hyun Kim, Ji Hye Park, Dong Phil Choi, Joo Young Lee, Hyeon Chang Kim

**Affiliations:** 1 Korean Human Resource Development Institute for Health & Welfare, Cheongju, Republic of Korea; 2 Department of Public Health, Yonsei University Graduate School, Seoul, Republic of Korea; 3 National Academy of Agricultural Science, Rural Development Administration, Jeonju, Korea; 4 Department of Preventive Medicine, Yonsei University College of Medicine, Seoul, Republic of Korea; 5 Cardiovascular and Metabolic Diseases Etiology Research Center, Yonsei University College of Medicine, Seoul, Republic of Korea; West Virginia University, UNITED STATES

## Abstract

**Introduction:**

Increasing evidence suggests that secondhand smoke exposure (SHSE) may affect not only physical health, but also mental health. Therefore, we evaluated the association between SHSE and depressive symptoms among Korean adolescents.

**Methods:**

The JS High School Study enrolled 1071 high school freshmen from a rural community of South Korea. The current analysis was limited to 989 adolescents (495 male and 494 female adolescents), after excluding 48 ever-smokers, 3 students with physician-diagnosed depression, and 31 students who did not complete the depression questionnaire. SHSE was assessed using a self-reported questionnaire and was classified into three groups: none, occasional exposure, and regular exposure. Depressive symptoms were assessed according to the Beck Depression Inventory (BDI) score, ranging from 0 to 63, and the presence of depressive symptoms was defined as a BDI score ≥10.

**Results:**

Overall, adolescents with SHSE were more likely to have depressive symptoms than those without SHSE (p = 0.042).In a sex-specific analysis treating the BDI score as a continuous variable, regular SHSE was independently associated with higher BDI scores in male adolescents (β = 2.25, p = 0.026), but not in female adolescents (β = 1.11, p = 0.253). Compared to no SHSE, the odds ratio for having depressive symptoms among male adolescents with regular SHSE was 2.17 (95% confidence interval, 1.11 to 4.25) after adjusting for age, body mass index, and study year, and 3.65 (95% confidence interval, 1.52 to 8.73) after adjusting for age, body mass index, study year, exercise, and household income.

**Conclusion:**

Regular exposure to secondhand smoke was associated with having depressive symptoms among Korean male adolescents.

## Introduction

The Centers for Disease Control and Prevention reported that there is no risk-free level of secondhand smoke exposure (SHSE) [1, 2].Over half of all children in the United States experience SHSE at home, in cars, or in public places [2]. Based on Korea Youth Risk Behavior Web-based Survey Statistics, 32.8% of male adolescents and 34.9% of female adolescents are exposed to second-hand smoke in their own homes [3][3]. According to the CDC, more than 300,000 children in the United States suffer each year from infections caused by SHSE, including bronchitis, pneumonia, and ear infections [2]. SHSE is also known to adversely affect the physical health of children and adolescents [4–7]. Meanwhile, increasing evidence suggests that SHSE exerts adverse effects on mental health [8–10].

Depression is one of the most common mental health problems, with an estimated 350 million people affected globally [11][11]. The World Health Organization has reported that prevalence of depression among adolescents around the world is about 5–10% [12][12]. Long-term depression of moderate or severe intensity can cause an affected person to suffer greatly and function poorly in daily life. Moreover, adolescent depression is an antecedent of many adverse outcomes in adulthood [13], and globally imposes a significant economic burden not only on individuals with the condition, but also on their families, communities, employers, and general government budgets [12, 14].

A few studies reported that SHSE can cause poor mental health, including depression; however, the effect of SHSE on depression has not been fully evaluated among Korean adolescents[8–10]. Accordingly, we assessed the association between SHSE and depressive symptoms, using data from a self-reported SHSE questionnaire and the Beck Depression Inventory (BDI), in a study of high-school students.

## Methods

### Study population

This study is a cross-sectional analysis, using baseline data from a study of Korean adolescents, namely the JS High School Study (JSHS) [15]. From 2007 to 2012, the JSHS enrolled 1071 high school freshmen in a rural community of South Korea. For the present analysis, we excluded 48 students who smoked more than 100 cigarettes in their lifetime, 3 students with psychiatrist-diagnosed depression, and 31 students who did not complete the BDI questionnaire. A total of 989 students (495 male and 494 female adolescents) was enrolled for analysis. Written informed consent was obtained from each participant and his/her parent or guardian. Informed consent forms were distributed to eligible students at least one week prior to the examination, so the participating students and their parents had enough time to understand the purpose and process of the study. On the day of examination, research staff checked whether each consent form was completed, and signed by the student as well as his/her parent or guardian. The study protocol, and consent procedure was approved by the Institutional Review Board of Severance Hospital at Yonsei University College of Medicine (Approval No. 4–20100169).

### Measurements

All students were interviewed individually using a self-reported questionnaire to obtain information about socio-demographic characteristics, health behaviors, medical history, and social-psychological stress. Physical activity was categorized into four groups based on frequency. Household income per month was divided into three groups: <3.0 million won, 3.0-<5.0 million won, or ≥5.0 million won. Parental education status was categorized into one of two groups: high school graduates and college graduates or higher. Frequency of SHSE was measured by a questionnaire, asking students how many times per week they experienced SHSE at home and/or school. Based on this questionnaire, students with SHSE were classified into three groups: no exposure, occasional exposure (1–2 times/week), and regular exposure (3–7 times/week).

Standing height was measured to the nearest 0.1 cm on a stadiometer, and body weight was measured to the nearest 0.1 kg on a digital scale (Seca 763; SECA, Hamburg, Germany) while wearing school uniforms. Body mass index (BMI) was calculated as weight in kilograms divided by the square of height in meters. Resting blood pressure was measured with an automated oscillometric device. An overnight fasting blood sample was collected after at least an 8-hour fast. Collected blood samples were analyzed at a central research laboratory for measurements of complete blood count, fasting glucose, and lipid profiles.

Depressive symptoms were assessed using the Beck Depression Inventory (BDI) questionnaire. The BDI consists of 21 questions for emotional, cognitive, motivational, physiological, and other symptoms. Each item contains four statements describing the intensity of depressive symptoms. Each item is rated on a scale from 0 to 3, reflecting how participants have felt over the past week. Thus, the total BDI scores range from 0 to 63, with higher scores representing greater disability. This index has demonstrated acceptable sensitivity and specificity in distinguishing between subjects with and without depressive symptoms, and is considered a valid and reliable measure of depressive symptoms [16, 17]. The presence of depressive symptoms was defined as a BDI score≥10, and participants were classified into three groups according to severity: normal (0 to 9), mild (10 to 15), and moderate to severe(16 to 63).

### Statistical analysis

General and clinical characteristics were described according to the status of SHSE, and their differences were assessed using an independent t-test and a chi-square test. Adolescents’ characteristics were also compared between those with depressive symptoms (BDI score≥10) and those without, and then compared again across BDI score groups (normal, mild, and moderate or greater depressive symptoms) using ANOVA test and chi-square test. Independent associations between SHSE and depressive symptoms were assessed using serial linear and logistic regression models: first model was unadjusted; second model was adjusted for age, BMI, and study year; and third model was additionally adjusted for exercise frequency and household income. All statistical analyses were performed using SAS software version 9.2 (SAS Inc., Cary, NC, USA). All analyses were two-sided, and *P*-values less than 0.05 were regarded as statistically significant.

## Results

General and clinical characteristics were shown by the status of SHSE in Table 1. Overall, adolescents who experienced SHSE showed significantly higher mean BDI score than those who did not experience SHSE (8.7 versus 7.5, p = 0.001). The likelihood of having depressive symptoms (BDI score≥10) was also higher in those with SHSE than those without (35.6% versus 29.1%, p = 0.042).

**Table 1:** Characteristics of participants by the status of SHSE.

Variables	None SHSE (n = 632)	SHSE (n = 357)	p value
Age, years	15.5 ± 0.6	15.4 ± 0.5	0.003
BMI, kg/m^2^	21.7 ± 3.0	21.8 ± 3.1	0.634
SBP, mmHg	110.1 ± 13.1	109.6 ± 13.4	0.608
DBP, mmHg	61.0 ± 7.5	61.0 ± 7.8	0.994
Fasting blood sugar, mg/dl	86.8 ± 7.0	88.0 ± 7.2	0.012
Total cholesterol, mg/dl	156.5 ± 26.9	153.7 ± 25.4	0.112
BDI score	7.5 ± 5.3	8.7 ± 6.1	0.001
Sex			
Male	342 (54.1)	153 (42.9)	0.001
Female	290 (45.9)	204 (57.1)	
Physical activity (n = 958)			
None	124 (20.2)	70 (20.4)	0.633
<2 per week	52 (8.5)	34 (9.9)	
2-<4 per week	334 (54.4)	174 (50.6)	
≥4 per week	104 (16.9)	66 (19.2)	
House income, won (n = 723)			
<3.0 million	92 (19.7)	62 (24.2)	0.050
3.0 -<5.0 million	189 (40.5)	115 (44.9)	
≥5.0 million	186 (39.8)	79 (30.9)	
Depressive symptoms by BDI score			
Normal	448 (70.9)	230 (64.4)	0.042
Having depressive symptoms	184 (29.1)	127 (35.6)	

Data expressed as mean ± standard deviation or number (%). BMI, Body mass index; SBP, Systolic blood pressure; DBP, Diastolic blood pressure; BDI, Beck Depression Inventory; SHSE, Secondhand smoke exposure.

Table 2 presents the characteristics according to the presence of depressive symptoms in male and female adolescents, respectively. Male adolescents with depressive symptoms were more likely to be younger (p = 0.007), have higher total cholesterol (p = 0.004), and exercise less frequently (p = 0.018) than those without depressive symptoms. SHSE was associated with depressive symptoms in male adolescents with borderline significance (p = 0.051).When comparing female to male adolescents with depressive symptoms, female adolescents reported higher rates of SHSE compared with male adolescents (28.5% and 23.3% for occasional SHSE and 16.4% and 13.0% for regular SHSE, respectively). However, among female adolescents, no covariates were significantly associated with depressive symptoms.

**Table 2:** Characteristics according to depressive symptoms status in 495 male adolescents and 494 female adolescents.

Male adolescents (n = 495)	Normal (n = 349)	Having depressive symptoms (n = 146)	p value
Age, years	15.5 ± 0.5	15.4 ± 0.5	0.007
BMI, kg/m^2^	22.2 ± 3.4	22.1 ± 3.3	0.787
SBP, mmHg	115.2 ± 12.7	116.4 ± 13.5	0.365
DBP, mmHg	61.3 ± 7.7	62.4 ± 8.0	0.142
Fasting blood sugar, mg/dl	88.0 ± 7.2	88.8 ± 7.2	0.288
Total cholesterol, mg/dl	146.3 ± 22.7	154.2 ± 29.0	0.004
Physical activity			
None	38 (11.1)	28 (19.9)	0.018
<2 per week	47 (13.7)	15 (10.6)	
2-<4 per week	168 (49.1)	74 (52.5)	
≥4 per week	89 (26.0)	24 (17.0)	
House income, won			
<3.0 million	52 (20.1)	31 (30.1)	0.101
3.0 -<5.0 million	103 (39.8)	39 (37.9)	
≥5.0 million	104 (40.2)	33 (32.0)	
SHSE			
None	249 (71.4)	93 (63.7)	0.051
Occasional	77 (22.1)	34 (23.3)	
Regular	23 (6.6)	19 (13.0)	
Female adolescents (n = 494)	Normal (n = 329)	Having depressive symptoms (n = 165)	p value
Age, years	15.4 **±** 0.6	15.4 ± 0.5	0.315
BMI, kg/m^2^	21.2 ± 2.5	21.3 ± 2.7	0.752
SBP, mmHg	104.1 ± 10.4	104.8 ± 11.7	0.499
DBP, mmHg	60.2 ± 7.4	60.7 ± 7.3	0.450
Fasting blood sugar, mg/dl	86.1 ± 6.9	86.6 ± 6.8	0.417
Total cholesterol, mg/dl	163.0 ± 26.3	161.2 ± 25.6	0.473
Physical activity			
None	86 (27.2)	42 (26.4)	0.294
<2 per week	18 (5.7)	6 (3.8)	
2-<4 per week	180 (57.0)	86 (54.1)	
≥4 per week	32 (10.1)	25 (15.7)	
House income, won			
<3.0 million	40 (16.8)	31 (25.2)	0.114
3.0 -<5.0 million	114 (47.9)	48 (39.0)	
≥5.0 million	84 (35.3)	44 (35.8)	
SHSE			
None	199 (60.5)	91 (55.2)	0.462
Occasional	78 (23.7)	47 (28.5)	
Regular	52 (15.8)	27 (16.4)	

Data expressed as mean ± standard deviation or number (%). BMI, Body mass index; SBP, Systolic blood pressure; DBP, Diastolic blood pressure; SHSE, Secondhand smoke exposure.

When BDI scores were further classified into three groups according to severity(normal, mild, and moderate to severe), frequency of regular SHSE increased correspondingly with depression severity both in male (6.6%, 11.4% and 17.1%, respectively) and female adolescents (15.8%, 16.2% and 16.7%, respectively). However, the difference was not statistically significant, probably owing to the small sample size (data presented in S1 Table). When SHSE was classified according to place of exposure (at home or at school), only SHSE at home in male adolescents was associated with depressive symptoms (p = 0.022 for dichotomized groups, p = 0.032 for three groups; data presented in S2 Table).

Table 3 describes the sex-specific association between SHSE and BDI when treating the scores as continuous variables. Regular SHSE in male adolescents was significantly associated with higher BDI score after adjusting for age, BMI, and study year, compared to those with no SHSE (β = 1.78, p = 0.035). This association became stronger with further adjustment for exercise frequency and household income (β = 2.25, p = 0.026). Although regular SHSE in female adolescents was associated with higher BDI score in the fully adjusted model compared to those with no SHSE, the association was not significant.

**Table 3:** Sex-specific association between SHSE and BDI score.

SHSE status	No.	BDI score	Unadjusted		Adjusted for age, BMI, and study year		Adjusted for age, BMI, study year, physical activity, and household income	
		Mean ± SD	ß (95% CI)	p	ß (95% CI)	p	ß (95% CI)	p
Among male adolescents	495							
None	342	7.1 ± 5.0	Ref		Ref		Ref	
Occasional	111	7.8 ± 4.9	0.64 (-0.46 to 1.74)	0.255	0.32 (-0.77 to 1.40)	0.570	-0.36 (-1.63 to 0.91)	0.575
Regular	42	8.9 ± 6.5	1.78 (0.13 to 3.43)	0.035	1.60 (-0.03 to 3.22)	0.054	2.25 (0.27 to 4.23)	0.026
Among female adolescents	494							
None	290	8.0 ± 5.5	Ref		Ref		Ref	
Occasional	125	9.2 ± 6.2	1.26 (0.01to 2.51)	0.049	1.08 (-0.18 to 2.34)	0.093	0.54 (-0.97 to 2.05)	0.482
Regular	79	9.1 ± 7.0	1.09 (-0.40 to 2.57)	0.152	0.68 (-0.86 to 2.23)	0.387	1.11 (-0.80 to 3.02)	0.253

SHSE, Secondhand smoke exposure; BDI, Beck Depression Inventory; BMI, Body mass index; 95% CI: confidence interval

Table 4 shows the sex-specific associations between SHSE and depressive symptoms. Male adolescents with regular SHSE had significantly higher odds ratio for having depressive symptoms (OR 2.07, 95% CI 1.06 to 4.01), compared to those with no SHSE. This association became stronger when adjusting for age, BMI, study year, exercise frequency, and household income, with an odds ratio of 3.65 (95% CI 1.52 to 8.73). In unadjusted analysis, when stratified by place of exposure, male adolescents with SHSE at home had a higher odds ratio for having depressive symptoms, however, only regular SHSE was significantly associated with depressive symptoms. The association between regular SHSE and depressive symptoms remained after adjusting for age, BMI, study year, exercise frequency, and household income, with an odds ratio of 4.43 (95%1.52–12.90). In female adolescents, no amount of SHSE was significantly associated with depressive symptoms. After being stratified by place of exposure, female adolescents with SHSE at home had a higher odds ratio for depressive symptoms, but no statistically significant association was observed in unadjusted or adjusted analyses.

**Table 4:** Sex-specific association between SHSE by site of exposure and depression.

SHSE by site of exposure	No. total	No. having depressive symptoms	Odds ratio (95% CI) for having depressive symptoms		
			Unadjusted	Adjusted for age, BMI, and study year	Adjusted for age, BMI, study year, physical activity, and household income
***Among male adolescents***					
SHSE, overall					
None	342	93	1.00	1.00	1.00
Occasional	111	34	1.08 (0.67–1.74)	1.05 (0.65–1.70)	0.85 (0.45–1.57)
Regular	42	19	2.07 (1.06–4.01)	2.17 (1.11–4.25)	3.65 (1.52–8.73)
SHSE at home					
None	386	104	1.00	1.00	1.00
Occasional	74	26	1.39 (0.81–2.37)	1.39 (0.81–2.38)	1.31 (0.66–2.61)
Regular	31	15	2.36 (1.11–5.01)	2.45 (1.15–5.25)	4.43 (1.52–12.90)
SHSE at school					
None	435	128	1.00	1.00	1.00
Occasional	36	9	0.72 (0.32–1.62)	0.70 (0.31–1.56)	0.43 (0.14–1.37)
Regular	12	5	1.66 (0.50–5.52)	1.79 (0.54–5.98)	3.29 (0.83–13.03)
***Among female adolescents***					
SHSE, overall					
None	290	91	1.00	1.00	1.00
Occasional	125	47	1.27 (0.81–1.98)	1.27 (0.81–1.98)	0.96 (0.56–1.66)
Regular	79	27	1.06 (0.61–1.85)	1.05 (0.61–1.83)	1.13 (0.57–2.23)
SHSE at home					
None	374	119	1.00	1.00	1.00
Occasional	84	31	1.25 (0.76–2.06)	1.25 (0.76–2.06)	1.07 (0.58–1.95)
Regular	33	15	1.62 (0.78–3.35)	1.60 (0.77–3.32)	1.70 (0.67–4.34)
SHSE at school					
None	362	121	1.00	1.00	1.00
Occasional	63	21	0.90 (0.50–1.61)	0.90 (0.50–1.61)	0.78 (0.40–1.51)
Regular	53	14	0.65 (0.33–1.28)	0.64 (0.32–1.28)	0.65 (0.29–1.47)

SHSE, Secondhand smoke exposure; BDI, Beck Depression Inventory; BMI, Body mass index

## Discussion

We observed a positive association between SHSE and depressive symptoms in Korean male adolescents. Exposure to tobacco smoke among young people has detrimental health effects, including respiratory complications [4, 6, 7, 18], metabolic syndrome [5], and mental problems [8–11, 19]. A previous study using US National Health and Nutrition Examination Survey data reported that SHSE was positively associated with symptoms of major depressive disorder, generalized anxiety disorder, attention-deficit/hyperactivity disorder, and conduct disorder in male children and adolescents [10]. These results are consistent with our findings, in that there was a significant association between SHSE and depression in male adolescents. A previous Korean study demonstrated that current smoking and SHSE are positively associated with depression in male and female adolescents[9]. A Scottish study reported that higher salivary cotinine levels were associated with psychological distress in children, especially for hyperactivity and conduct disorder [8]. In a study of non-smoking adolescents in Hong Kong, SHSE was associated with poor academic performance [20]. On the contrary, a study of multi-ethnic adolescents in Chicago Public Schools reported that high salivary cotinine levels were inversely associated with depressive symptoms in non-smoking students [21]. A Dutch study found no evidence that plasma cotinine levels were related to either depressive or anxiety symptoms in non-smokers [22].

There are several possible explanations for the association of SHSE with depressive symptoms. First, secondhand smoke itself can be stressful to non-smoking children and adolescents. Regular SHSE at home or outside is a chronic stressor, and such chronic stress may lead to the development of depressive symptoms [23].Another mechanism might involve the dopaminergic system, which is known to be related to the risk of depression [24]. Animal studies observed that tobacco smoke has an acute and long-term effect on the dopamine system. A mouse study found that exposure to tobacco smoke elevates dopamine D1 and D2 receptors in the brains of rats [25]. Another mouse study has shown that exposure to secondhand smoke impacts c-aminobutyric acid b2 receptors (GABAB2), dopamine transporter mRNA expression, and dopamine receptors [26]. Other animal studies also report that nicotine and particulate matter in tobacco smoke may lead to long-term imbalances in dopamine transport [27]. Nicotine exposure induced a negative mood and decreased mobility in rats [28].Another biological mechanism that may link SHSE to depression is chronic inflammation [29, 30]. Many studies have proposed that activation of inflammatory cytokines plays a role in the development of depression [31–34]. Cytokines induce enzyme indoleamine2, 3-dioxygenase, which limits tryptophan and serotonin transporter and may cause depression [34].

Even though smoke-free legislation has contributed to a successful decline in SHSE among adolescents, adolescents living with smokers continue to be exposed to tobacco smoke [35, 36]. Additionally, many studies report that early-life exposure to tobacco smoke exerts harmful effects on the mental health of children and adolescents throughout their entire life [37–39]. Around 5–10% of adolescents are affected by depression, and this mental problem imposes an important economic burden, not only on individuals requiring treatment, but also on their families and communities due to a consequent loss of capacity as a member of society [12, 14, 40]. Prevention of depression, by monitoring and modifying exposure to secondhand smoke, could contribute to the decrease of deaths and injuries arising from suicide and the related economic burden.

The present study has some limitations. First, as a cross-sectional study, in which all information was gathered at the same point in time, we could not establish the causal relationship between SHSE and depressive symptoms. It may be difficult to affirm whether SHSE leads to depressive symptoms or is a result of such symptoms. Second, we measured the degree of SHSE and depressive symptoms using an interviewer-assisted questionnaire. There is a possibility of misclassification bias in measuring SHSE and depressive symptoms. Measurement of SHSE is unlikely to be differential to BDI scores. The BDI has been found to have high internal consistency (α = 0.88) and test-retest reliability (*r* = 0.60) in previous reports [17]. Thus, our findings are unlikely to be severely distorted by measurement error. Third, we were unable to assess the effects of duration and intensity of SHSE because we did not measure number of smokers in the household, smoking duration of smokers, or exposure duration of study participants. Lastly, our study population was limited to students from a single rural area; therefore, our findings may not be generalizable to other regions.

## Conclusions

We found a significant association between SHSE and depressive symptoms among male adolescents, and in particular, SHSE at home was strongly associated with depressive symptoms. Further studies are needed to clarify the relevant biological or psychological mechanisms, as well as potential reasons for differential associations across sex strata.

### Ethics approval

The study protocol, and consent procedure was approved by the Institutional Review Board of Severance Hospital at Yonsei University College of Medicine (Approval No. 4–20100169).

## Supporting Information

S1 Dataset This file is dataset analyzed for the manuscript. (XLSX)

S1 Table Characteristics according to depressive symptoms status in 495 male adolescents and 494 female adolescents. (DOCX)

S2 Table Sex-specific distribution of SHSE by site of exposure according to depressive symptoms status. (DOCX)
