# Supplementary material for: Secondhand Smoke Exposure and Depressive Symptoms among Korean Adolescents: JS High School Study
Source: PLoS One. 2016 Dec 30;11(12):e0168754. doi: 10.1371/journal.pone.0168754 (PMC5201244; doi:10.1371/journal.pone.0168754)
Supplement: S1 Table — (DOCX) [file pone.0168754.s002.docx]

S1 Table. Characteristics according to depressive symptoms status in 495 male adolescents and 494 female adolescents

| Male adolescents (n = 495) | Normal (n = 349) | Mild (n = 105) | p^a^ | Moderate to severe (n = 41) | p^b^ |
| --- | --- | --- | --- | --- | --- |
| Age, years | 15.5 ± 0.5 | 15.4 ± 0.5 | 0.091 | 15.3 ± 0.5 | 0.006 |
| BMI, kg/m^2^ | 22.2 ± 3.4 | 22.3 ± 3.4 | 0.779 | 21.6 ± 2.9 | 0.284 |
| SBP, mmHg | 115.2 ± 12.7 | 117.5 ± 13.0 | 0.116 | 113.6 ± 14.3 | 0.447 |
| DBP, mmHg | 61.3 ± 7.7 | 63.1 ± 8.2 | 0.042 | 60.8 ± 7.2 | 0.680 |
| Fasting blood sugar, mg/dl | 88.0 ± 7.2 | 88.2 ± 7.4 | 0.859 | 90.3 ± 6.5 | 0.049 |
| Total cholesterol, mg/dl | 146.3 ± 22.7 | 155.8 ± 31.5 | 0.005 | 150.0 ± 21.0 | 0.320 |
| Physical activity |  |  |  |  |  |
| None | 38 (11.1) | 21 (20.2) | 0.029 | 7 (18.9) | 0.270 |
| <2 per week | 47 (13.7) | 13 (12.5) |  | 2 (5.4) |  |
| 2-<4 per week | 168 (49.1) | 54 (51.9) |  | 20 (54.1) |  |
| ≥4 per week | 89 (26.0) | 16 (15.4) |  | 8 (21.6) |  |
| House income |  |  |  |  |  |
| <3.0 million | 52 (20.1) | 19 (25.3) | 0.619 | 12 (42.9) | 0.010 |
| 3.0 -<5.0 million | 103 (39.8) | 28 (37.3) |  | 11 (39.3) |  |
| ≥5.0 million | 104 (40.2) | 28 (37.3) |  | 5 (17.9) |  |
| SHSE |  |  |  |  |  |
| None | 249 (71.4) | 68 (64.8) | 0.216 | 25 (61.0) | 0.055 |
| Occasional | 77 (22.1) | 25 (23.8) |  | 9 (22.0) |  |
| Regular | 23 (6.6) | 12 (11.4) |  | 7 (17.1) |  |
| Female adolescents (n = 494) | Normal (n = 329) | Mild (n = 111) | p^a^ | Moderate to severe (n = 54) | p^b^ |
| Age, years | 15.4 ± 0.6 | 15.4 ± 0.5 | 0.204 | 15.4 ± 0.5 | 0.974 |
| BMI, kg/m^2^ | 21.2 ± 2.5 | 21.2 ± 2.6 | 0.961 | 21.4 ± 2.9 | 0.579 |
| SBP, mmHg | 104.1 ± 10.4 | 104.8 ± 12.4 | 0.600 | 104.8 ± 10.2 | 0.637 |
| DBP, mmHg | 60.2 ± 7.4 | 60.7 ± 7.4 | 0.521 | 60.7 ± 7.1 | 0.613 |
| Fasting blood sugar, mg/dl | 86.1 ± 6.9 | 86.5 ± 7.1 | 0.568 | 86.8 ± 6.3 | 0.468 |
| Total cholesterol, mg/dl | 163.0 ± 26.3 | 159.3 ± 25.3 | 0.206 | 164.9 ± 25.9 | 0.615 |
| Physical activity |  |  |  |  |  |
| None | 86 (27.2) | 27 (25.2) | 0.600 | 15 (28.9) | 0.161 |
| <2 per week | 18 (5.7) | 6 (5.6) |  | 0 (0.0) |  |
| 2-<4 per week | 180 (57.0) | 58 (54.2) |  | 28 (53.9) |  |
| ≥4 per week | 32 (10.1) | 16 (15.0) |  | 9 (17.3) |  |
| House income |  |  |  |  |  |
| <3.0 million | 40 (16.8) | 20 (23.8) | 0.214 | 11 (28.2) | 0.235 |
| 3.0 -<5.0 million | 114 (47.9) | 32 (38.1) |  | 16 (41.0) |  |
| ≥5.0 million | 84 (35.3) | 32 (38.1) |  | 12 (30.8) |  |
| SHSE |  |  |  |  |  |
| None | 199 (60.5) | 65 (58.6) | 0.933 | 26 (48.2) | 0.162 |
| Occasional | 78 (23.7) | 28 (25.2) |  | 19 (35.2) |  |
| Regular | 52 (15.8) | 18 (16.2) |  | 9 (16.7) |  |

Data expressed as mean ± standard deviation or number (%)

BMI, Body mass index; SBP, Systolic blood pressure; DBP, Diastolic blood pressure; SHSE, Secondhand smoke exposure

a: Normal vs. mild

b: Normal vs. moderate or severe depressive symptoms
