# Supplementary material for: Secondhand Smoke Exposure and Depressive Symptoms among Korean Adolescents: JS High School Study
Source: PLoS One. 2016 Dec 30;11(12):e0168754. doi: 10.1371/journal.pone.0168754 (PMC5201244; doi:10.1371/journal.pone.0168754)
Supplement: S2 Table — (DOCX) [file pone.0168754.s003.docx]

S2 Table. Sex-specific distribution of SHSE by site of exposure according to depressive symptoms status

| SHSE status among males | Normal (n = 349) | Having depressive symptoms (n = 146) | | *p-value*  normal vs. having symptoms | *p-value*  across three categories |
| --- | --- | --- | --- | --- | --- |
|  |  | Mild (n = 105) | Moderate or severe (n = 41) |  |  |
| SHSE at home |  |  |  |  |  |
| None | 282 (81.5) | 74 (71.2) | 30 (73.2) | 0.022 | 0.032 |
| Occasional | 48 (13.9) | 21 (20.2) | 5 (12.2) |  |  |
| Regular | 16 (4.6) | 9 (8.7) | 6 (14.6) |  |  |
| SHSE at school |  |  |  |  |  |
| None | 307 (90.0) | 94 (92.2) | 34 (85.0) | 0.547 | 0.581 |
| Occasional | 27 (7.9) | 5 (4.9) | 4 (10.0) |  |  |
| Regular | 7 (2.1) | 3 (2.9) | 2 (5.0) |  |  |
| SHSE status among females | Normal (n = 329) | Having depressive symptoms (n = 165) | | *p-value*  normal vs. having symptoms | *p-value*  across three categories |
|  |  | Mild (n = 111) | Moderate or severe (n = 54) |  |  |
| SHSE at home |  |  |  |  |  |
| None | 255 (78.2) | 82 (73.9) | 37 (68.5) | 0.221 | 0.439 |
| Occasional | 53 (16.3) | 20 (18.0) | 11 (20.4) |  |  |
| Regular | 18 (5.5) | 9 (8.1) | 6 (11.1) |  |  |
| SHSE at school |  |  |  |  |  |
| None | 241 (74.8) | 84 (80.0) | 37 (72.6) | 0.592 | 0.671 |
| Occasional | 42 (13.0) | 12 (11.4) | 9 (17.7) |  |  |
| Regular | 39 (12.1) | 9 (8.6) | 5 (9.8) |  |  |

SHSE, Secondhand smoke exposure
